# A novel benzo-heterocyclic amine derivative N30 inhibits influenza virus replication by depression of Inosine-5’-Monophospate Dehydrogenase activity

**DOI:** 10.1186/s12985-017-0724-6

**Published:** 2017-03-15

**Authors:** Jin Hu, Linlin Ma, Huiqiang Wang, Haiyan Yan, Dajun Zhang, Zhuorong Li, Jiandong Jiang, Yuhuan Li

**Affiliations:** Institute of Medicinal Biotechnology, Chinese Academy of Medical Sciences and Peking Union Medical College, Beijing, China

**Keywords:** Benzo-heterocyclic amine derivative, IMPDH, Influenza A virus

## Abstract

**Backgroud:**

Influenza virus is still a huge threat to the world-wide public health. Host inosine-5’- monophosphate dehydrogenase (IMPDH) involved in the synthesis of guanine nucleotides, is known to be a potential target to inhibit the replication of viruses. Herein, we evaluated antiviral activity of a benzo-heterocyclic amine derivative N30, which was designed to inhibit IMPDH.

**Results:**

The results demonstrated that N30 inhibited the replication of H1N1, H3N2, influenza B viruses, including oseltamivir and amantadine resistant strains in vitro. Mechanistically, neuraminidase inhibition assay and hemagglutination inhibition assay suggested that N30 did not directly target the two envelope glycoproteins required for viral adsorption or release. Instead, the compound could depress the activity of IMPDH type II. Based on these findings, we further confirmed that N30 provided a strong inhibition on the replication of respiratory syncytial virus, coronavirus, enterovirus 71 and a diverse strains of coxsackie B virus.

**Conclusions:**

We identified the small molecule N30, as an inhibitor of IMPDH, might be a potential candidate to inhibit the replication of various viruses.

## Background

Influenza A virus (IAV) is extremely prone to cause periodic epidemics and pandemics in the world through evolution by point mutations or swapping of gene segments, correspondingly [1–3].

At present, vaccination and antiviral drugs are principle strategies to prevent influenza. Available anti-influenza drugs include inhibitors of neuraminidase (NA) (e.g. oseltamivir and zanamivir) [4], M2 proton channel (amantadine and rimantadine) [5], and RNA-dependent RNA polymerase (RdRp) (favipiravir) [6]. However, influenza vaccines must be reformulated each year due to the constant antigenic evolution of influenza. Additionally, NA inhibitors and M2 ion-channel inhibitors have limited efficacies as drug resistance occurrence [7, 8], and they only worked at the early phase of virus infection. The inherent property of influenza viruses to mutate, resulting in low efficacy of available drugs, has underscored the necessity of developing alternative strategies to provide protection against pandemic influenza.

Widespread utilization of directly antiviral drugs accelerates resistance problem, hence host cellular factors become attractive therapeutic targets to treat influenza virus infections. Recent studies were pioneered in host purine metabolic pathway, a conserved process responsible for providing host cells with a ready supply of guanosine triphosphate (GTP) for critical cellular processes [9]. Host cells could produce GTP either in de novo pathway or salvage pathway. While in the de novo synthesis of GTP, IMPDH catalyzes the oxidation of inosine monophosphate (IMP) to xanthosine monophosphate (XMP) which is the rate-limiting step. Growing evidences support that inhibition of IMPDH decreases intracellular levels of guanosine nucleotides in DNA or RNA synthesis, thereby indirectly inhibits virus replication which requires host guanine nucleotides as raw materials [10, 11]. As IMPDH inhibitors targeted on host cells, it would be less susceptible for selection of drug-resistant strains.

In early study, we established a series of novel benzo-heterocyclic amine derivatives and determined their in vitro antiviral activities. Notably, compounds 3d (N30) showed potent activity towards IAV at low micromolar concentrations [12]. In the present study, we developed the broad-spectrum antiviral activity of N30 in vitro, including oseltamivir-resistant strains and amantadine-resistant strains of influenza virus, coxsackie B virus, coronavirus, and respiratory syncytial virus. In addition, we examined the inhibitory rate of the compound against two IAV envelope glycoproteins, hemagglutinin and neuraminidase, and investigated its effect on expression and the enzyme activity of IMPDH type II. Our results indicated that N30 is a potential compound with antiviral activities through suppressing the activity of IMPDH type II, these finding also proves that development of anti-influenza drugs directing at IMPDH is warranted.

## Methods

### Compounds

**Fig. 1:**
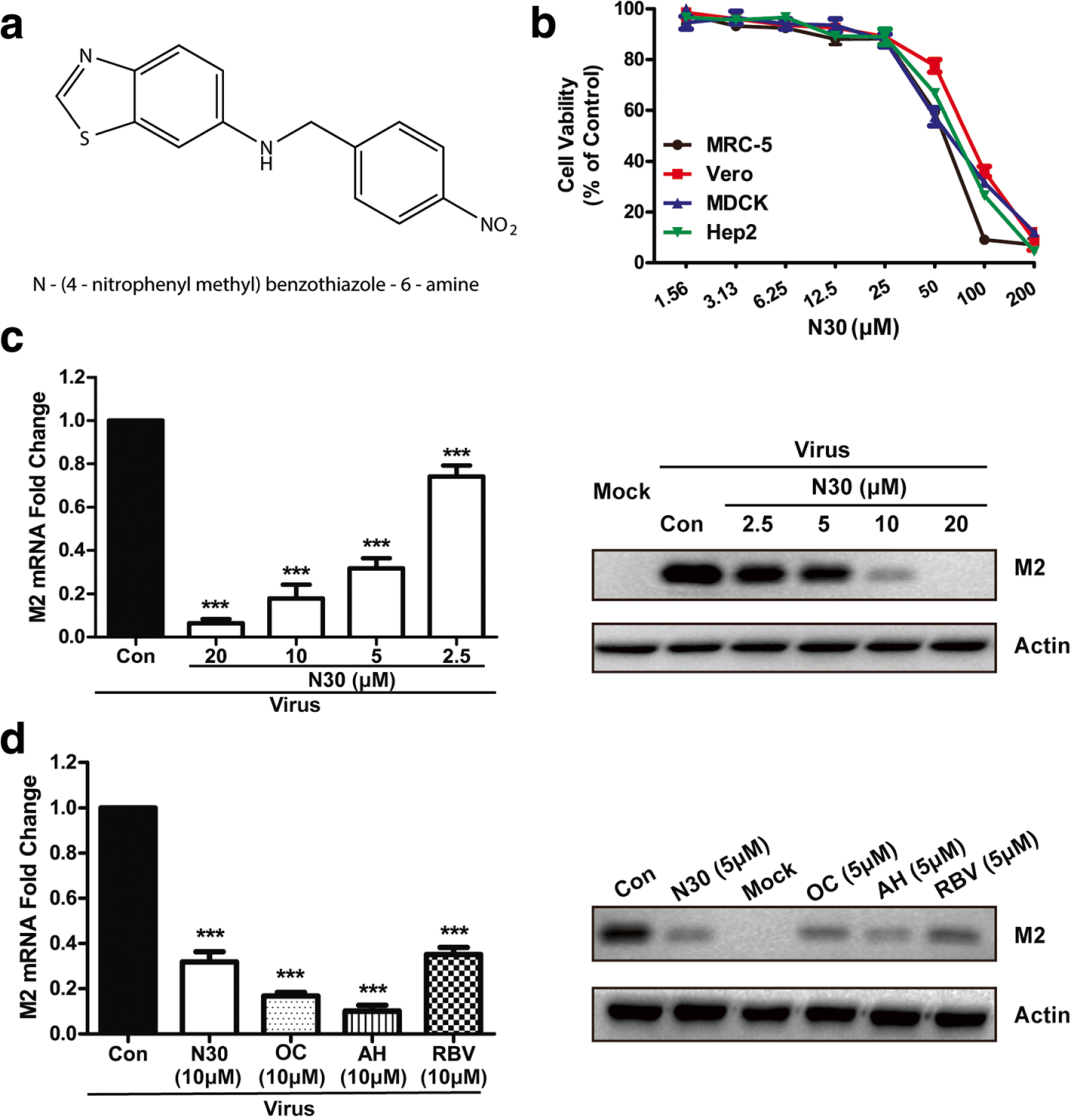
Effects of N30 on IAV M2 protein and RNA expression in MDCK. **a** Chemical structure of N30. **b** The effect of N30 on viabilities of MRC-5, Vero, MDCK and Hep2 cells, which were measured by CCK. **c** The effect of N30 with different concentrations on IAV M2 protein and RNA expression. **d** The effect of N30 on IAV M2 protein and RNA expression compared with reference drugs. MDCK cells were infected with IAV A/Fort Monmouth/1/1947(H1N1) (0.01 multiplicity of infection [MOI, plaque-forming units (PFU)/cell]) for 2 hours, and treated with different concentrations of N30 or reference drugs for 24 h. Total mRNA and protein were extracted at 24 h post-infection, and analyzed by qRT-PCR and Western blotting respectively. Mock: normal cells without treatment; Con: infected cells treated with equal amounts of DMSO as N30. The experiments were performed in triplicate, and the data represents mean ± SD. ***P < 0.001 versus Con (ANOVA)

N30 (N-(4-nitrophenyl methyl) benzothiazole-6-amine, Fig. 1a) was originally provided by Professor Zhuorong Li at the Institute of Medicinal Bioechnology Chinese Academy of Medical Sciences, Beijing, China. Pirodavir (Biochempartner), oseltamivir carboxylate (OC, Medchem), amantadine hydrochloride (AH, Sigma-Aldrich) and Ribavirin (RBV, Sigma-Aldrich) were used as reference drugs in vitro. N30, pirodavir and AH were dissolved in dimethyl sulfoxide (DMSO, Sigm-Aldrich). OC and RBV were dissolved at phosphate buffered saline (PBS, Gbico).

### Culture of cells

Cells in this study were all purchased from the America Type Culture Collection (ATCC). Madin-Darby canine kidney (MDCK) cells were grown in Minimum Essential Medium (MEM, Invitrogen) containing 1% Non-Essential Amino Acids Solution (NEAA, Invitrogen), 1% Penicillin-Streptomycin (10,000 U/mL) (Invitrogen) and 10% fetal bovine serum (FBS, Gbico). African green monkey kidney (Vero) cells and Human lung embryonated cells (MRC-5) cells were cultured in MEM supplemented with 1% antibiotics and 10% or 15% FBS respectively. While Hep2 cells were grown in DMEM / F12 (1:1) (Invitrogen) supplemented with 1% antibiotics and 10% FBS. Cells were cultured at 37 °C in the presence of 5% CO_2_.

### Viruses and virus infection

Influenza A/Fort Monmouth/1/1947 (H1N1) strain was perchased from ATCC. Clinical isolated strains A/TianjinJinnan/15/2009 (H1N1, oseltamivir-resistant), A/LiaoningZhenxing/1109/2010 (H1N1, oseltamivir-resistant), A/Wuhan/359/1995 (H3N2), A/FujianTongan/196/2009 (H3N2, amantadine-resistant), A/HunanZhuhui/1222/2010 (H3N2, amantadine-resistant), BV/Shenzhen/155/2005 and BY/FujianXinluo/54/2006 were kindly provided by Professor Yuelong Shu at the Institute for Viral Disease Control and Prevention, China Centers for Disease Control and Prevention. These strains were all obtained by propagating in 10-day-old embryonated chicken eggs for 48 or 72 h.

Coxsackie virus B (CVB) strains CVB1 (strain Conn-5) and CVB5 (strain Faulkner) were kindly provided by Professor Zhaohua Zhong, Department of Microbiology, Harbin Medical University. Enterovirus 71 (EV71) strain SZ98 was kindly provided by Dr. Qi Jin, Institute of Pathogen Biology, Chinese Academy of Medical Science and Peking Union Medical School, Beijing, China. EV71 strain BrCr, CVB strains CVB2 (strain Ohio-1), CVB3 (strain Nancy), CVB4 (strain J.V.B.), CVB6 (strain Schmitt), respiratory syncytial virus (RSV) strain (long strain) and human coronavirus (229E) were all obtained from ATCC. EV71 virus and CVB virus were propagated in Vero cells, RSV, and coronavirus were propagated in Hep2 and MRC-5 cells respectively.

For influenza virus infections, MDCK cells were washed with PBS and infected with influenza virus diluted in serum-free medium at 37 °C for 2 h. Then, the viral inoculum was replaced by maintenance medium supplemented with 2 μg/ml TPCK-treated trypsin (Worthington) and 0.08% BSA (Sigma-Aldrich). As for other virus infections, virus was diluted in serum-free medium and incubated with cells at 37 °C for 1 h. Then, the viral inoculum was replaced by maintenance medium supplemented with 2% FBS.

### Cytotoxicity test

The cytotoxicity of N30 in MDCK, Vero, Hep2, and MRC-5 was evaluated by Cell Counting Kit (CCK, Transgen Biotech). Briefly, cells were seeded in 96-well culture plates (MDCK cells were 2.5 × 10^4^ per well, Hep2 cells were 1 × 10^4^ per well, Vero and MRC-5 cells were 4 × 10^4^ per well). 16 h later, cells were incubated with serial two-fold dilutions of N30 for another 48 h. Then, 10 μL of CCK was added to cells, the absorbance was read at 450 nm on Enspire (Perkin Elmer) after 4 hours incubation. The TC_50_ (defined as the 50% toxicity concentration of drugs) were determined by Reed and Muench method [13].

### Cytopathic effect (CPE) assays

The attached cells were incubated with virus (100 TCID_50_) in serum-free medium for 2 h at 37 °C. The unconjugated viruses were replaced by maintenance medium with serial two-fold dilutions of N30 or positive control drugs. The reduction of virus-induced CPE were recorded when the CPE in viral control groups reached 100%, then according to Reed and Muench method, 50% cell-inhibitory concentrations (IC_50_) and selectivity index (SI, calculated as the ratio of TC_50_/IC_50_) of compounds were calculated [14].

### Western blot assay

Cells were collected at 24 h post-infection and lysed in M-PER mammalian protein extraction reagent containing halt protease inhibitor cocktail (Thermo Fisher Scientific), sample proteins were subjected to SDS-PAGE and transferred to PVDF membrane (Millipore). After blocked with 5% milk, the membranes were incubated with antibodies against Influenza M2, β-actin (Santa Cruz), CVB3 VP1 (Millipore), IMPDH type II (Sigma-Aldrich), RSV M2 (Santa Cruz) and coronavirus (FIPV-70, Santa Cruz) respectively, then, the HRP-conjugated secondary antibodies were incubated and the signals were detected using an enhanced chemiluminescence (ECL) kit (GE Healthcare Life Sciences).

### Quantitative real-time RT-PCR

Total RNA was extracted at 24 h post-infection and purified using the RNeasy Mini Kit (Qiagen). The one-step quantitative real-time RT-PCR was carried out using an ABI 7500 Fast real-time PCR instrument (Applied Biosystems) using SuperScript III Platinum SYBR Green One-Step qRT-PCR kit (Transgen) with the following conditions: 50 °C for 5 min, 95 °C for 30 seconds, followed by 35 cycles of 95 °C for 5 s, 60 °C for 30 s. The mRNA of IAV M2, CVB3 VP1, RSV M2, and GAPDH were amplified with specific primers (Oligonucleotides used were shown in Table 1) [15, 16]. The samples were normalized by subtracting the CT values of GAPDH and the relative amounts of viral RNA were calculated.

**Table 1:** Oligonucleotides used in this study

Oligonucleotide	Sequence
5’ M2 (influenza)	GACCRATCCTGTCACCTCTGAC
3’ M2 (influenza)	GGGCATTYTGGACAAAKCGTCTACG
5’β-actin (Monkey)	TGACGGGGTCACCCACACTGTGCCCATCTA
3’ β-actin (Monkey)	CTAGAAGCATTTGCGGTGGACGATG
5’ VP1 (CVB3)	TGCTCCGCAGTTAGGATTAGC
3’ VP1 (CVB3)	ACATGGTGCGAAGAGTCTATTGAG
5’ M2 (RSV)	GTTGCCATGAGCAAACTCCT
3’ M2 (RSV)	ACGTCTGCTGGCAATCTTTT
5’ N (CoV-229E)	CGCAAGAATTCAGAACCAGAG
3’ N (CoV-229E)	GGCAGTCAGGTTCTTCAACAA
5’ GAPDH (Homo)	GGTGGTCTCCTCTGACTTCAACA
3’ GAPDH (Homo)	GTTGCTGTAGCCAAATTCGTTGT

### NA inhibition assay

The inhibition activity were assessed by quantifying the fluorescent product of the enzymatic reaction upon the cleavage of 4-methylumbelliferyl-a-D-N-acetylneuraminic acid (MUNANA). Briefly, the 100 μL reaction system consisted of 20 μL sample, 20 μL enzyme and 60 μL substrate buffer mix (20 μM MUNANA, 33 mM MES buffer (pH 3.5), 4 mM CaCl_2_, double distilled water). 60 μL substrate buffer mix was added after incubating diluted drug samples and enzyme for 60 min at room temperature. The fluorescence intensity was read before and after incubating for 15 min on Enspire Multimode Reader (PerkinElmer), with excitation and emission wavelength were 355 nm and 460 nm respectively. The relative fluorescence were obtained by subtracting the background value [15].

### Hemagglutination inhibition (HI) assay

The inhibitory effect of compound N30 on viral attachment to target cells was assessed by HI assay using a 1.2% chicken red blood cell suspension. Briefly, the diluted IAV were mixed with diluted compounds in a U bottom 96-well plate. After incubation at 4 °C for 40 min, equal volume of 1.2% chicken erythrocyte suspension was added to each well. Then, the erythrocyte aggregation was evaluated by visual inspection after 40 min [17, 18].

### Enzymatic assay of IMPDH type II

The enzymatic assay was employed to evaluate the inhibitory effect of N30 on the activity of IMPDH type II by following the increase in formation of nicotinamide-adenine dinucleotide (NADH), which absorbance was read at 340 nm on Enspire at 30 °C. The 200 μL assay buffer contained 1 M Tris/HCl (pH 8.8), 1 M KCl, 30 mM EDTA, 10 μL IMPDH type II (Sigma-Aldrich), 1 mM DTT and NAD (Sigma-Aldrich). The reaction was started after addition of IMP, with final concentrations was 1 mM [19].

## Results

### Antiviral activity of N30 against influenza viruses in vitro

The cytotoxic effect of N30 on cell viabilities including MDCK, Vero, MRC-5 and Hep2 cells were measured with CCK, illustrated in Fig. 1b. According to the results of cytotoxicity, we selected non toxicity dose to implement subsequent experiments. Antiviral activity of N30 against influenza viruses were obtained by the method of CPE in MDCK cells, with RBV, OC and AH as reference drugs. IC_50_ and SI values of N30 against influenza viruses were shown in Table 2. N30 efficiently inhibited all tested strains of influenza A and B virus,including oseltamivir-resistant strains A/tianjinjinnan/15/2009, A/liaoningzhenxing/1109/2010, and amantadine-resistant strains A/fujiantongan/196/2009, A/hunanzhuhui/1222/2010.

**Table 2:** Inhibitory activity of N30 against eight influenza strains

Strain	N30		RBV		OC		AH	
	IC_50_(μM)	SI	IC_50_(μM)	SI	IC_50_(μM)	SI	IC_50_(μM)	SI
A/FortMonmouth/1/1947	1.93 ± 0.16	34.54	1.64 ± 0.72	>121.95	0.25 ± 0.01	>200	0.65 ± 0.59	>307.69
A/tianjinjinnan/15/2009	1.67 ± 0.49	39.92	2.54 ± 1.25	>78.74	2.57 ± 0.10	>19.45	18.24 ± 0.24	>10.96
A/liaoningzhenxing/1109/2010	3.43 ± 3.36	19.44	4.92 ± 3.02	>40.65	3.20 ± 0.21	>15.58	10.13 ± 0.04	>19.74
A/wuhan/359/1995	1.31 ± 0.87	50.89	8.23 ± 6.31	>24.30	0.12 ± 0.01	>416.67	15.64 ± 0.31	>12.78
A/fujiantongan/196/2009	1.13 ± 0.43	59.00	4.42 ± 0.74	>45.25	0.28 ± 0.01	>178.57	78.84 ± 3.04	>2.54
A/hunanzhuhui/1222/2010	1.76 ± 0.24	37.89	2.34 ± 0.98	>85.47	0.78 ± 0.02	>64.10	57.24 ± 2.23	>3.49
BV/shenzhen/155/2005	1.16 ± 0.69	57.47	1.70 ± 0.57	>117.65	1.02 ± 0.08	>49.02	>200	-
BY/fujianxinluo/54/2006	0.70 ± 0.18	95.24	2.48 ± 0.53	>80.65	0.56 ± 0.02	>89.28	>200	-

Note: The TC_50_ of N30, OC, RBV and Amantadine were 66.67 μM, >50 μM, >200 μM, >200 μM, respectively

“−”: no antiviral activity at the maximal nontoxic concentration

Antiviral activity of N30 were also demonstrated in the results of western blot and real-time qPCR, which displayed the dosage dependent relationship of N30 in reducing the amounts of IAV A/Fort Monmouth/1/1947 (H1N1) M2 protein and RNA in MDCK cells (Fig. 1c and d). To sum up, N30 demonstrated a potent antiviral activities against IAV H1N1, H3N2, and influenza B viruses,including drug-resistant strains.

### N30 does not inhibit the functions of NA or HA

**Fig. 2:**
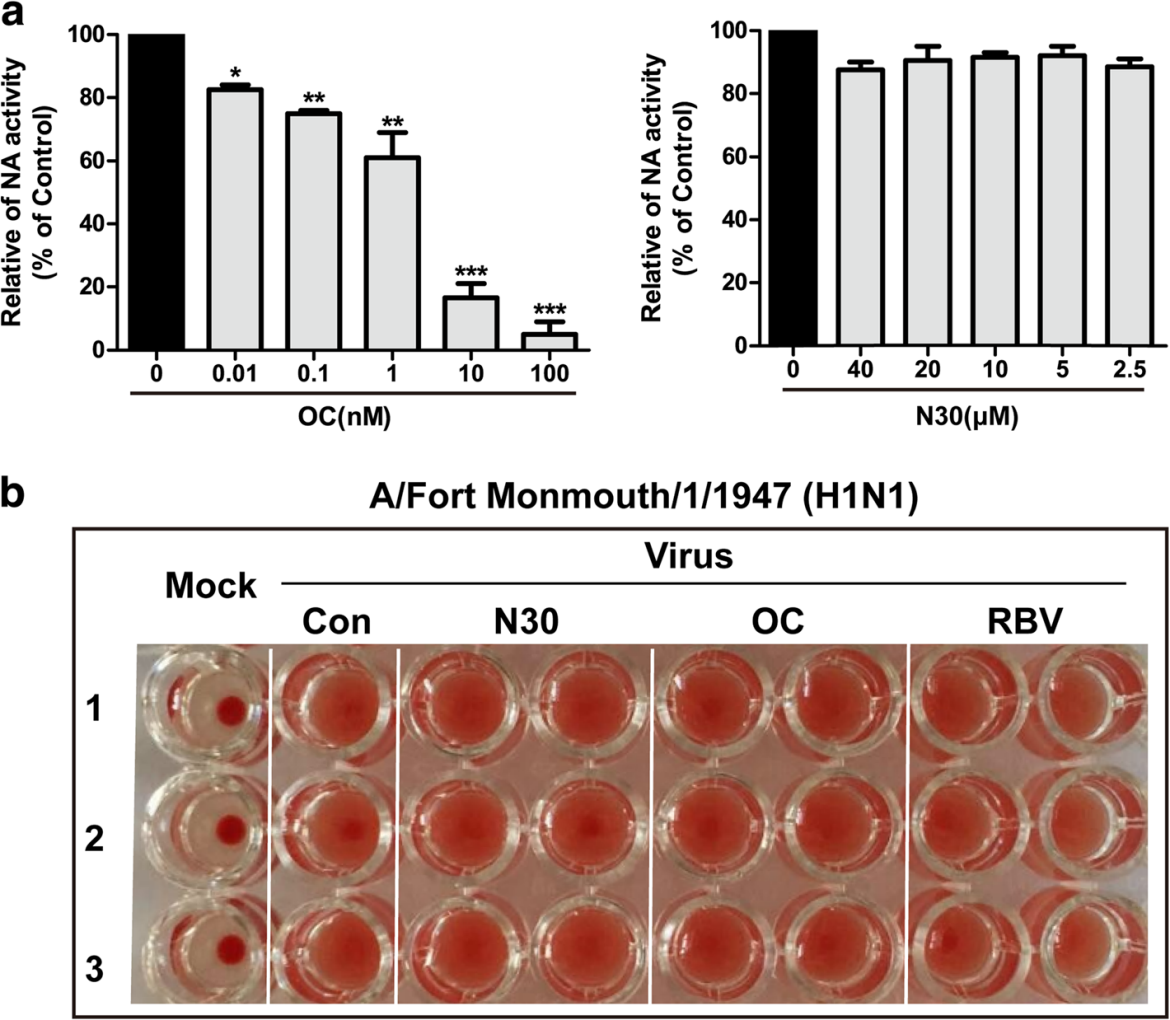
N30 does not inhibit the functions of the two IAV envelope glycoproteins. **a** The effect of OC and N30 on IAV NA activity. **b** The effect of N30 or references drugs on hemagglutination inhibition. The maximum concentration (in row 1) of N30, OC and RBV were 40 μM. A serial of 10-fold dilution for N30 and reference drugs were adopted in the following rows. The experiments were performed in triplicate, and each value represents mean ± SD. *P < 0.05, **P < 0.01, ***P < 0.001

NA and HA are two critical envelope glycoproteins of influenza virus. NA inhibition assay was performed to identify any effect of N30 on influenza virus release. As shown in Fig. 2a. N30 had little inhibitory effect on the NA activity of IAV A/FM1/1947,while OC,the reference drug,could significantly reduce the activity of NA in a dose dependent manner.

Furthermore, as displayed in Fig. 2b, N30 could not inhibit the aggregation of chicken erythrocytes caused by viral hemagglutinin binding to sialic acid receptors on the cellular membrane surface, while negative control drugs RBV or OC also did not show the inhibitory effect as they are not targeted on HA [1, 20]. The results suggested N30 probably does not target on two IAV envelope glycoproteins required for virus adsorption or release, the antiviral effect of N30 is more likely attribute to inhibiting one or multiple intracellular replication process of influenza virus.

### N30 inhibits IAV replication by inhibiting IMPDH type II activity instead of reducing IMPDH expression

**Fig. 3:**
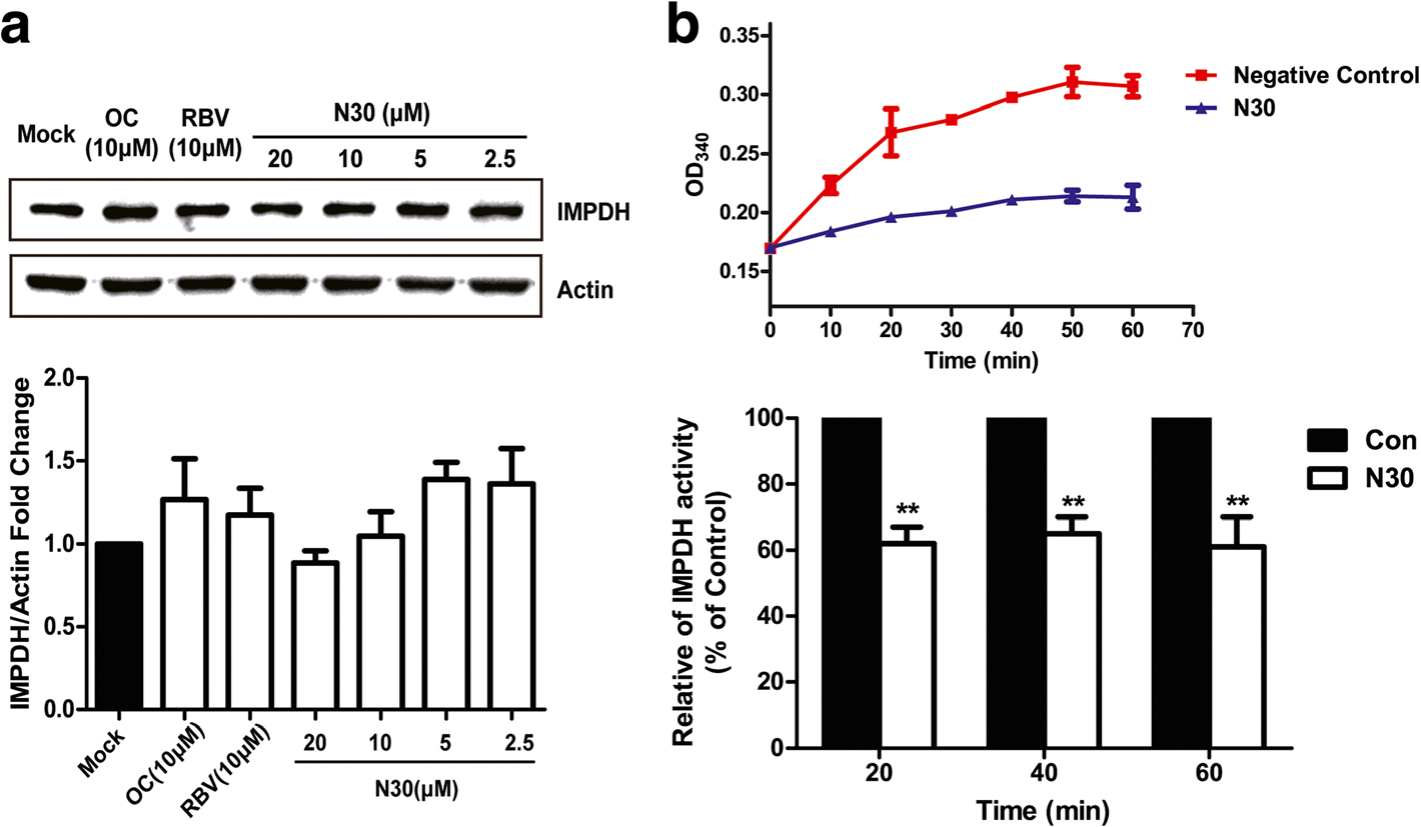
N30 does not inhibit the protein expression but inhibit the enzymatic reaction of IMPDH. **a** The effect of N30 on IMPDH type II expression. MDCK cells were treated with RBV, OC or serial dilutions of N30 for 24 h before intracellular protein extraction. The protein expression of IMPDH was detected by western blot. **b** The effect of N30 on IMPDH type II activity. The experiments were performed in triplicate, and each value represents mean ± SD

Ribavirin had been demonstrated to possess a broad activity against several RNA and DNA viruses, including influenza A and B virus [21], measles virus, Parainfluenza virus and so on [22–24]. IMPDH had been demonstrated to play a critical role in viral suppression of RBV-MP, the phosphorylated form of RBV. Accumulating evidence showed that depression of IMPDH inhibits replication of diverse species of viruses. N30 was synthesized targeting on IMPDH, so we further studied the effect of N30 on the enzyme. The results showed that IMPDH expressions have not been lessened after N30 treatment, even RBV had no inhibition either. On the other hand, the enzymatic reaction rate of IMPDH could be reduced by 38.18% after N30 treatment. Hence, the anti-influenza activity of N30 might be related to the inhibition the IMPDH-driven enzymatic reaction (Fig. 3).

### The broad spectrum of N30 antiviral activity in vitro

Extensive pharmacological activity of IMPDH inhibitors has been researched and some has been applied in clinic. Considering the products of IMPDH catalyzed reaction are necessary for viral replication, N30 should also exhibit inhibitory effects on other viruses. In support of this hypothesis, we tested the ability of N30 to protect cells from infection of virus, such as EV71, CVB, RSV and coronavirus.

**Fig. 4:**
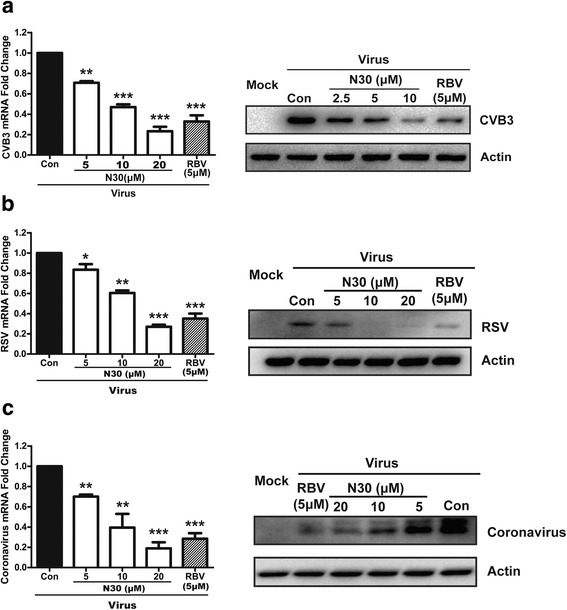
Inhibitory activities of N30 against CVB3, RSV and coronavirus. **a** The effect of N30 on CVB3 VP1 protein and mRNA expression in Vero cells. N30 was added to Vero cells after the cells were infected with Coxsackie B virus (0.005 MOI) for 2 hours, CVB3 VP1 mRNA and protein were extracted at 24 h post-infection. **b** The inhibitory effect of N30 on expression of RSV M2 mRNA and protein in Hep2 cells. N30 was added to Hep2 cells simultaneously with RSV (0.025 MOI) infection. RSV M2 mRNA and protein were extracted at 24 h post-infection. **c** The effect of N30 on coronavirus. N30 was added to MRC-5 cells after infection with coronavirus (0.005 MOI) for 2 hours. Total cellular RNA and proteins was collected 24 h after infection. All the experiments were performed with 5 μM RBV or DMSO as control. The experiments were performed in triplicate, and the data represents mean ± SD. *P < 0.05, **P < 0.01, ***P < 0.001 versus Con

As exhibited in Tables 3 and 4, N30 showed antiviral activities against different strains of EV71 (reference drug pirodavir) or CVB (reference drug RBV). In addition, N30 suppressed CVB3 structural protein 1 (VP1) mRNA and protein expressions in a dose dependent manner (Fig. 4a).

**Table 3:** Inhibitory activity of N30 against different strains of Cosackie B virus

Strain	N30			RBV		
	TC_50_ (μM)	IC_50_ (μM)	SI	TC_50_ (mM)	IC_50_ (mM)	SI
CVB1	89.56 ± 4.25	6.28 ± 1.61	14.26	>20.00	2.37 ± 0.36	>8.44
CVB2	89.56 ± 4.25	11.65 ± 2.94	7.69	>20.00	2.27 ± 0.18	>8.81
CVB3	89.56 ± 4.25	1.82 ± 2.76	49.21	>20.00	3.13 ± 1.21	>6.39
CVB4	89.56 ± 4.25	10.37 ± 3.13	8.64	>20.00	2.47 ± 1.60	>8.10
CVB5	89.56 ± 4.25	2.54 ± 1.43	35.26	>20.00	1.20 ± 0.53	>16.67
CVB6	89.56 ± 4.25	6.81 ± 1.89	13.15	>20.00	4.56 ± 2.41	>4.39

**Table 4:** Inhibitory activity of N30 against different strains of Human enterovirus 71

Strain	N30			Pirodavir		
	TC_50_ (μM)	IC_50_ (μM)	SI	TC_50_ (μM)	IC_50_ (μM)	SI
EV71(SZ98)	89.56 ± 4.25	10.63 ± 3.76	8.42	32.57 ± 0.55	0.27 ± 0.27	120.6
EV71(BrCr)	89.56 ± 4.25	8.04 ± 3.77	11.14	32.57 ± 0.55	0.16 ± 0.00	203.56

Next, we evaluated the inhibitory action of N30 on RSV and Coronavirus replication. As presented in Fig. 4b, N30 markedly decreased the RSV M2 mRNA and protein expressions in RSV-infected Hep2 cells, Moreover, 10 μM N30 treatment almost completely inhibit the expression of the viral proteins. Additionally, a similar suppression of N30 towards coronavirus was displayed in Fig. 4c.

Taken together, our data identified N30 possessed a broad spectrum antiviral activity against different strains of CVB and EV71, RSV and coronavirus in vitro.

### N30 showed little toxicity in vivo on survival rate or body weight in mice

To determine the in vivo toxicity of N30 preliminarily, female Kun Ming mice were gavaged 100 mg/kg and 50 mg/kg N30 twice a day. Six mice enrolled in each group were gavaged with N30 or vehicle for continuous administration for 14 days, then weighted at day 0, 5, 10 and 14 respectively, shown in Table 5. The body weight changes of mice were observed in groups. We found there were no significant differences between the N30 group and vehicle-treated control group (5% CH_3_CH_2_OH + 5% Castor oil was applied as the parallel solvent control.). This indicates that the dose of 100 mg/kg by oral administration in mice had little acute toxicity.

**Table 5:** Effects of N30 on body weight in mice

Days	Body weight (g)			
	N30 (100 mg/kg)	N30 (50 mg/kg)	Solvent control	Control
0	17.0 ± 1.1	17.0 ± 1.3	16.0 ± 0.9	15.9 ± 0.7
5	23.7 ± 0.9	21.6 ± 1.0	22.0 ± 1.9	22.7 ± 1.3
10	28.5 ± 2.1	26.3 ± 0.9	26.2 ± 2.3	27.6 ± 1.0
14	30.1 ± 2.5	27.7 ± 1.5	27.9 ± 2.6	29.9 ± 1.6

## Discussion

For a long time, infection with numerous different viruses has brought prodigious threat to human health. Influenza viruses display considerable antigenic diversity, and new strains arising from antigenic mutation always cause pandemics over the world. Therefore, a key challenge is searching for novel drug targets with broad antiviral spectrum and difficult to generate viral resistance. As we know, viruses always use a host protein or signaling pathway to complete its replication, therefore host factors are attractive therapeutic targets to treat virus infections.

IMPDH controls the conversion of IMP to XMP, which is the rate-limiting step in de novo synthesis of guanine nucleotides [25]. Since guanine, as the raw material in replication of virus, needs to be synthesized by host cells, inhibition of IMPDH could restrain virus replication eventually. Even if an organism or tissue can produce guanine nucleotides in salvage pathways through several phosphoribosyl transferases [26], the amount of guanine nucleotides can not meet the need for rapidly growing cells or viruses. So, the replication of influenza virus could be inhibited through downregulating guanine nucleotides synthesis by inhibiting the activity of IMPDH. IMPDH activity is determined by two highly conserved isoforms type I and type II in human and mammalian cells [27], which show different biological functions [28]. The comparation of N30 on the two subtypes of IMPDH is worth doing in the future.

In our study, we demonstrated the antiviral effect of N30 against influenza virus, including resistant strains against oseltamivir and amantadine. We have excluded the target of neuraminidase or hemagglutinin and found its antiviral mechanism may involve the inhibition of guanine nucleotides synthesis by inhibiting the activity of IMPDH type II. As IMPDH is necessary for viral replication, the antiviral effect of N30 on EV71, CVB, RSV and coronavirus has been confirmed. In addition, N30 showed little toxicity in vivo on survival rate or body weight in mice, and we will further detect the antiviral efficacy of N30 in vivo. Based on the above results, we believe that studying N30 or other IMPDH inhibitors has prospect future in antiviral research.

Although many IMPDH inhibitors have been developed, their biological function and mechanism are still needed to be studied. In addition, the current application of IMPDH inhibitors has some side effects. In early studies on influenza virus-infected patients, RBV administered orally elevated the bilirubin values. This may reflect the destruction of the RBV-containing erythrocytes. In Lassa fever infected patients, when treated orally or intravenously with higher doses of RBV, a transient anemia was also observed [29]. Besides, the side effects limited the use of mycophenolate mofetil (MMF, another IMPDH inhibitor) either, such as diarrhea, leucopenia, sepsis, and vomiting, and adverse effects on fetal researches occurred in experimental animals [30]. These highlight an urgent need to reduce the toxicity of IMPDH inhibitors. Therefore, we will perform more extensive laboratory studies on N30 in pharmacokinetics, pesticide effect, and toxicity in vivo to provide a theoretical basis for its druggability.

## Conclusion

N30 inhibited the replication of H1N1, H3N2, influenza B viruses, including oseltamivir and amantadine resistant strains in vitro. N30 did not directly target the two envelope glycoproteins required for viral adsorption or release. Instead, the compound could depress the activity of IMPDH type II. N30 provided a strong inhibition on the replication of respiratory syncytial virus, coronavirus, enterovirus 71 and a diverse strains of coxsackie B virus.

## Abbreviations

IMPDH: Inosine-5’-Monophospate Dehydrogenase
IAV: Influenza A virus
OC: Oseltamivir carboxylate
AH: Amantadine hydrochloride
RBV: Ribavirin
CVB: Coxsackie virus B
EV71: Enterovirus 71
RSV: Respiratory syncytial virus
